# A Prospective Multicenter Standard of Care Study of Outpatient Laparoscopic Sleeve Gastrectomy

**DOI:** 10.1007/s11695-024-07094-8

**Published:** 2024-02-17

**Authors:** Amit Surve, Daniel Cottam, Aurora Pryor, Samuel Cottam, Robert Michaelson, Thomas Umbach, Michael Williams, Hossein Bagshahi, Laura July, Racquel Bueno, Devorah Chock, Matthew Apel, Christopher Hart, William Johnson, Brendon Curtis, Amy Rosenbluth, Konstantinos Spaniolas, Walter Medlin, Whitney Wright, Ciara Lee, Christy Lee, Rachael Trujeque, Deborah Rinker

**Affiliations:** 1 Bariatric Medicine Institute, 1046 E 100 S, Salt Lake City, UT USA; 2 Stony Brook University Hospital, 23 South Howell Ave, Centereach, NY USA; 3 Northwest Weight & Wellness Center, 125 130Th St SE, Everett, WA USA; 4 Blossom Bariatrics, 7385 S Pecos Rd #101, Las Vegas, NV USA; 5 Atlanta General and Bariatric Surgery Center, 6300 Hospital Parkway Ste. 150, Johns Creek, GA USA; 6 HB Health PLLC, 1101 W Rosedale St Suite 1, Fort Worth, TX USA

**Keywords:** Prospective multicenter study, Standard of care study, Outpatient laparoscopic sleeve gastrectomy, Ambulatory setting, Same-day surgery center, Registry

## Abstract

**Abstract:**

A global shift is occurring as hospital procedures move to ambulatory surgical settings. Surgeons have performed outpatient sleeve gastrectomy (SG) in bariatric surgery since 2010. However, prospective trials are needed to ensure its safety before widespread adoption.

**Purpose:**

The study aimed to present a comprehensive report on the prospective data collection of 30-day outcomes of outpatient primary laparoscopic SG (LSG). This trial seeks to assess whether outpatient LSG is non-inferior to hospital-based surgery in selected patients who meet the outpatient surgery criteria set by the American Society for Metabolic and Bariatric Surgery.

**Materials and Methods:**

This study is funded by the Society of American Gastrointestinal and Endoscopic Surgeons and has been approved by the Advarra Institutional Review Board (Pro00055990). Cognizant of the necessity for a prospective approach, data collection commenced after patients underwent primary LSG procedures, spanning from August 2021 to September 2022, at six medical centers across the USA. Data centralization was facilitated through ArborMetrix. Each center has its own enhanced recovery protocols, and no attempt was made to standardize the protocols.

**Results:**

The analysis included 365 patients with a mean preoperative BMI of 43.7 ± 5.7 kg/m^2^. Rates for 30-day complications, reoperations, readmissions, emergency department visits, and urgent care visits were low: 1.6%, .5%, .2%, .2%, and 0%, respectively. Two patients (0.5%) experienced grade IIIb complications. There were no mortalities or leaks reported.

**Conclusion:**

The prospective cohort study suggests that same-day discharge following LSG seems safe in highly selected patients at experienced US centers.

**Graphical Abstract:**

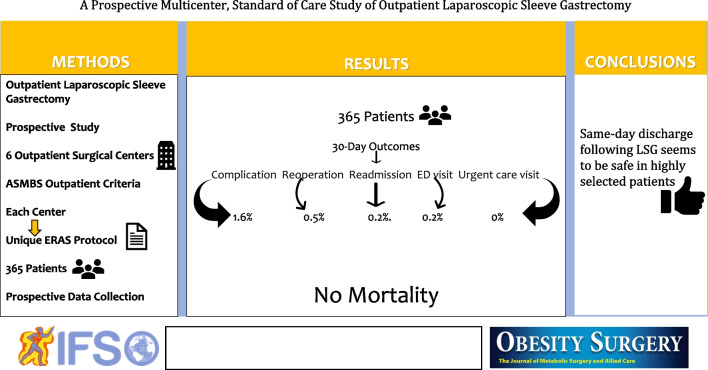

Minimal-invasive surgery (MIS) has emerged as a promising surgical technique due to its advantages over traditional open surgery [1]. The application of MIS techniques has revolutionized the field of bariatrics by significantly reducing postoperative pain, recovery time, and hospital stays while simultaneously improving cosmetic outcomes and cost-effectiveness. The technological advancements in MIS have provided bariatric surgeons with a novel way of performing procedures and enabled them to perform elective procedures on an outpatient basis. As a result, MIS has gained widespread acceptance and has become an essential part of the standard of care for bariatric surgical procedures.

Minimally invasive techniques have become widely accepted and commonly practiced in bariatric surgical procedures. Moreover, a notable surge in the utilization of outpatient procedures has been observed in the USA, where a significant proportion of surgical interventions are now conducted employing minimally invasive techniques within outpatient surgical settings. This paradigm shift signifies a shift towards less invasive approaches in bariatric surgery and underscores the increasing adoption of outpatient settings for such procedures [2, 3]. Furthermore, implementing enhanced recovery after surgery (ERAS) protocols has added further benefits in the context of MIS [4].

Outpatient procedures typically require less hospitalization and are more cost-effective, making them an appealing choice for many patients and third-party payors in obesity surgery [5–7]. As a result, bariatric surgery centers are increasingly adopting outpatient procedures to meet the needs of their patients while maintaining high-quality care. Numerous well-established bariatric procedures, including laparoscopic adjustable gastric banding (LAGB), laparoscopic sleeve gastrectomy (LSG), laparoscopic Roux-en-Y gastric bypass (LRYGB), and laparoscopic single anastomosis duodenal switch with sleeve (SADI-S), are now performed as outpatient surgeries with a stay of less than 24 h [3, 8–10]. These procedures are performed in ambulatory centers, considering the technical complexity involved, which can vary from simple to complex operations [3, 8–10]. Of these procedures, LSG accounts for 61.6% of all bariatric procedures, both inpatient and outpatient [11]. Although retrospective studies have shown favorable outcomes for LSGs when performed in an outpatient setting, it is imperative to conduct prospective outcome trials to investigate the safety of this approach thoroughly [3, 12–23].

The study is a non-inferiority investigation designed to prospectively assess the safety of LSG when performed in an outpatient surgery center, adhering to the American Society for Metabolic and Bariatric Surgery (ASMBS) outpatient criteria [24]. This assessment involves the examination of postoperative 30-day mortality, morbidity, readmission, and reoperation rates.

## Methods

The present study is financially supported by a grant ($280,00) provided by the Society of American Gastrointestinal and Endoscopic Surgeons (SAGES) and has been approved by the Advarra Institutional Review Board (Pro00055990).

In this multi-institutional (six centers across the USA) study, data were gathered from the medical records of 365 patients who had undergone primary laparoscopic sleeve gastrectomy by 19 surgeons from August 2021 through September 2022 and were anticipated discharged on the same day of surgery. Each patient met the criteria outlined by the ASMBS for outpatient surgery.

A list of centers in the USA known for performing outpatient SGs was compiled. The lead research team contacted these institutions and provided comprehensive study details, including the necessary institutional review board (IRB) paperwork and study design. The decision to participate was at each center’s discretion, considering factors such as available staff and resources, as additional personnel were required for data entry.

The selection of centers was based on identifying institutions with a track record of proficiency in performing outpatient SGs. Each center was given the autonomy to engage experienced surgeons from their teams to ensure a diverse group of institutions. This approach aimed to capture a representative sample of institutions performing outpatient SG while also mitigating the potential introduction of selection bias. It is worth noting that no effort was made to enforce a standardized ERAS protocol across the centers. Instead, each experienced center was allowed to maintain its unique approach.

The study population consisted of ambulatory patients aged 18–65 with a body mass index (BMI) of < 55 kg/m^2^ for males and < 60 kg/m^2^ for females. Patients were excluded from the study if they had undergone prior bariatric surgery, were pregnant or lactating, exhibited end-organ failure, or demonstrated significant cardiac conditions (myocardial infarction, unstable angina, decompensated heart failure, high-grade arrhythmias, or hemodynamically significant valvular heart disease). Additionally, exclusion criteria encompassed pulmonary impairment such as end-stage lung disease or severe obstructive sleep apnea, candidacy for an organ transplant, preoperative weight exceeding 450 lbs, anticipated operative time exceeding 2 h, expression of a preference for an alternative bariatric procedure, and belonged to a vulnerable population (intellectually challenged, substance use, justice-involved individuals), or an estimated life expectancy of fewer than 6 months (terminally ill). These criteria mimic the ASMBS requirement for outpatient bariatric surgery (24).

All participating centers in the study adhered to an informed consent process that explained the procedure, associated risks, and potential benefits before the surgery. Furthermore, each center adhered to its individualized standard of care protocol to ensure careful patient selection and strict adherence to their respective ERAS protocol.

Patients arrived at the ambulatory surgical center on the day of surgery. Patients were discharged home from the ambulatory surgical center on the same day. Early and continuous ambulation was encouraged throughout their stay. At discharge, they received standard postoperative care unique to each participating center. Each center had a unique postoperative protocol for patient safety, including DVT risk mitigation, postoperative follow-up schedule, dehydration prevention, and nausea protocols.

Preoperative and 30-day postoperative data were collected into each center’s electronic medical record (EMR). Data from each center were prospectively entered into the electronic data capture (EDC) software designed for the present study by ArborMetrix, Inc. In the data acquisition process, dedicated personnel, typically one to two individuals per center, were responsible for data collection and entry utilizing the EDC system devised explicitly for this study. Upon completing data entry for each case, a lead research study coordinator conducted a meticulous review to ensure precision and completeness, identifying and rectifying any absent or inaccurate data points. Instances of identified errors prompted the creation of a detailed spreadsheet outlining the discrepancies for individual patients. Subsequently, the assigned data entry staff addressed these errors under the supervision of the coordinator, who re-evaluated the case and provided approval upon satisfactory correction.

During the data analysis phase, any identified errors were promptly communicated to the respective centers, prompting immediate corrective action. Strict adherence to predefined study protocol rules was enforced throughout the data collection and entry procedures. Instances of protocol non-compliance triggered immediate notification to the concerned centers, accompanied by reminders to uphold protocol adherence and minimize the potential for procedural errors.

The data collection for each patient was started after surgery. Demographic information, medical history, and physical exam results were collected during screening visits, including age, sex, ethnicity, race, weight, height, and BMI, along with particular attention to obesity-related comorbidities such as hypertension (HTN), gastroesophageal reflux disease (GERD), obstructive sleep apnea (OSA), hyperlipidemia (HLD), and diabetes mellitus (DM). All postoperative 30-day complications, transfer to an inpatient setting, emergency room (ER) visits, readmissions, reoperations, or death-related information were recorded with the date of discovery.

The study endpoints were established to evaluate the safety of LSG when performed on an ambulatory basis. Thirty-day adverse event rates measured safety endpoints, including transfer to inpatient, ER visits, urgent care visits, readmission, and reoperation rates. These endpoints were chosen to evaluate the short-term safety of the procedure. Adverse events were defined as any undesirable event within 30 days of surgery and were classified according to the Clavien-Dindo grading system [3, 25]. The primary outcome measure was the rate of adverse events, and the secondary outcome measures were the individual safety endpoints. All adverse events were recorded and reported according to established reporting guidelines.

The statistical analysis employed in this study aimed to provide a robust evaluation of the safety outcomes. Continuous variables were characterized using means and standard deviations, while categorical variables were characterized using frequencies and percentages. The statistical methods used for the analysis were chosen to ensure the reliability and validity of the findings. In addition, a meticulous data curation and cleaning process was implemented to enhance the accuracy and integrity of the dataset. This process involved systematically examining and validating the collected data to identify and rectify any inconsistencies, errors, or outliers. The curation process aimed to improve the overall quality of the data, ensuring that it accurately represented the patient population and outcomes under investigation.

## Results

A total of 365 patients were included in the analysis, of which 84.3% were female and 15.6% were male. The preoperative mean weight was 267.1 ± 43.5 lbs (121.1 ± 19.7 kg), and the mean BMI was 43.7 ± 5.7 kg/m^2^. The prevalence of preoperative comorbidities and treatment among the patients is shown in Table 1. The most prevalent preoperative comorbidity was HTN (33.9%), and the majority of patients with HTN were taking anti-hypertensive medication (96.7%). The second most prevalent comorbidity was GERD (27.9%), and most patients with GERD were taking medications for it (91.7%). Other comorbidities included OSA (21.9%), HLD (21.9%), and DM (14.5%) (Table 1). Most patients with OSA were prescribed CPAP/BiPAP (97.5%), and most used it (92.2%). However, over half of the patients reported that OSA interfered with their daily activities (65%).

**Table 1:** Demographics

Variable	Value
Patient (*n*)	365
M/F (%)	15.6/84.3
Weight (lbs)*	267.1 ± 43.5
BMI (kg/m^2^)*	43.7 ± 5.7
IBW (lbs) *	137.7 ± 17.3
EBW (lbs) *	129.4 ± 36
Preoperative co-morbidity and treatment	
1. HTN (*n* [%])	124 (33.9)
a. Medication (*n* [%])	120 (96.7)
2. GERD (*n* [%])	102 (27.9)
a. Medication (*n* [%])	93 (91.7)
3. OSA (*n* [%])	80 (21.9)
a. CPAP/BIPAP prescribed (*n* [%])	78 (97.5)
b. CPAP/BiPAP used (*n* [%])	71 (92.2)
c. Interferes with daily activity (*n* [%])	52 (65)
4. HLD (*n* [%])	80 (21.9)
a. Medication (*n* [%])	63 (78.7)
5. DM (*n* [%])	53 (14.5)
a. Medication (*n* [%])	48 (94.1)

^*^Values expressed as mean ± standard deviation

Abbreviation: *BMI* body mass index, *IBW* ideal body weight, *EBW* excess body weight; *n* number of patients, *HTN* hypertension, *GERD* gastroesophageal reflux disease, *OSA* obstructive sleep apnea, *HLD* hyperlipidemia, *DM* diabetes mellitus

Of the 365 patients who underwent the surgical procedure, a majority (92.6%) received a laparoscopic approach, and 7.3% received a robotic approach (Table 2). Of all the patients, 83.5% had an ASA score of III, 15.6% had a score of II, and only 0.2% had a score of I. No patient in the study had an ASA score of IV or higher. VTE prophylaxis was administered to all patients, with 83.5% receiving pharmacological and mechanical prophylaxis and only 6.5% receiving mechanical prophylaxis only. In the majority of patients (66.8%), the Medtronic gastrointestinal anastomosis (GIA) stapler was used (Table 2). A concurrent procedure was performed in 12% of patients.

**Table 2:** Intraoperative and early surgical outcomes

Variable	Value
ASA score	
a. I (*n* [%])	1 (.2)
b. II (*n* [%])	57 (15.6)
c. III (*n* [%])	305 (83.5)
d. IV, V, VI	0
VTE prophylaxis	
a. Mechanical only (*n* [%])	24 (6.5)
b. Pharmacological and mechanical (*n* [%])	305 (83.5)
Surgical approach	
a. Laparoscopic (*n* [%])	338 (92.6)
b. Robotic (*n* [%])	27 (7.3)
GIA Stapler	
a. Ethicon (*n* [%])	3 (.8)
b. Medtronic (*n* [%])	244 (66.8)
c. Other (n [%])	118 (32.3)
Concurrent procedure (*n* [%])	44 (12)
Total operative time (skin to skin [min. sec.]) *	52.8 ± 21.5
Leak test (*n* [%])	243 (66.5)
Drain placed (*n* [%])	30 (8.2)
Intraoperative complication (*n*)	0
Conversion to another approach (n)	1 (.2%)
Length of stay [hr. min.])	7.46 ± 3.40
30-day follow-up (%)	100
30-day reoperation (*n* [%])	2 (.5)
30-day readmission (*n* [%])	1 (.2)
30-day ER visit (*n* [%])	1 (.2)
30-day urgent care visit (*n*)	0
Death (*n*)	0

^*^Value expressed as mean ± standard deviation

Abbreviation: *ASA* American Society of Anesthesiologists, *n* = number of patient, *VTE* = venous thromboembolism, *GIA* = gastrointestinal anastomosis, *ER* = emergency room

The mean total operative time (skin-to-skin) was 52.8 ± 21.5 min (Table 2). Leak tests were performed in 66.5% of patients, and drains were placed in 8.2% of patients. No intraoperative complications were reported. One (0.2%) patient required conversion to another surgical approach. The mean length of stay was 7 h 46 min. ± 3 h 40 min.

All patients had a 30-day follow-up. In the first 30 days after the surgery, 0.5% required reoperation, 0.2% required readmission, and 0.2% required ER visits. No patients required urgent care visits. No deaths were reported during the follow-up period.

In total, six patients (1.6%) experienced short-term complications, as shown in Table 3. Three patients (0.8%) experienced grade I complications according to the Clavien-Dindo classification, two patients (0.5%) experienced grade IIIb complications, and one patient (0.2%) experienced a grade II complication. The most common complication was constipation, which was observed in two patients (0.5%). One patient (0.2%) encountered an intra-abdominal hemorrhage on the day of surgery, while another patient (0.2%) developed a recurrent hiatal hernia, which was repaired during the primary SG surgery 10 days prior to the sleeve migrating into the patient’s thoracic cavity. One patient (0.2%) experienced dehydration, and one patient (0.2%) experienced nausea.

**Table 3:** Short-term complication

Complication	No	Clavien-Dindo classification grade				
		I (no.)	II (no.)	IIIa (no.)	IIIb (no.)	V (no.)
Constipation	2	2				
Intra-abdominal hemorrhage*	1				1	
Hiatal hernia^@^	1				1	
Dehydration	1		1			
Nausea	1	1				
Total (no. [%])	6 (1.6%)	3 (.8%)	1 (.2%)	0	2 (.5%)	0

^*^Same-day complication. ^@^Recurrent hiatal hernia and ER visit

Clavien-Dindo classification grade: I, any deviation from the normal postoperative course; II, normal course altered; IIIa, complications that require an intervention performed under local anesthesia; IIIb, complications that require an intervention performed under local anesthesia; V, death

Abbreviation: *no.* number of events

## Discussion

Outpatient SG is gaining popularity [14]. Our study found that outpatient LSG is a safe and viable option for eligible patients. The rates of 30-day reoperation, readmission, ER visit, urgent care visit, morbidity, and mortality were found to be consistent with the findings reported in existing literature, encompassing both retrospective outpatient studies and prospective inpatient hospital-based studies. These findings support the increased utilization of outpatient LSG as an alternative to inpatient LSGs, particularly for patients who prefer the convenience and cost-effectiveness of an outpatient setting [26, 27].

In the extant literature, the major concerns associated with outpatient procedures involve postoperative complications, the insufficiency of patient monitoring upon discharge, the likelihood of insufficient pain management, and the necessity of patient education and adherence to postoperative instructions. Further apprehensions have been raised regarding the potential inadequacy of follow-up care and early discharge, allowing insufficient recuperation time after the procedure. Thus, debates continue about the feasibility and safety of outpatient SG and the importance of patient selection and postoperative care.

The concerns associated with outpatient SG in the literature highlight the need for appropriate patient selection and postoperative management. However, using the ERAS protocol has been pivotal in promoting favorable surgical outcomes for outpatient SG [3, 28]. ERAS is a comprehensive, multimodal approach to perioperative care that aims to reduce surgical stress, minimize complications, and accelerate recovery [29]. The protocol includes intraoperative strategies such as anesthesia and postoperative interventions such as early mobilization, optimized pain management, and a standardized diet plan. By implementing ERAS, patients undergoing SG experience less postoperative pain, nausea, and fatigue and have shorter hospital stays with a faster resumption of regular activities [3]. ERAS is particularly advantageous for outpatient SG, as it improves patient outcomes and reduces the risk of readmission. As such, implementing ERAS in outpatient SG can effectively address the concerns surrounding postoperative complications, insufficient monitoring, and early discharge, resulting in successful surgical outcomes. However, there needs to be agreement in the literature on what the best ERAS protocol is.

Instead of imposing a uniform ERAS protocol on each center, we chose to allow flexibility, enabling each center to implement the ERAS protocol that suited them best. Our belief was that this approach would not yield significant differences in outcomes, and our findings supported this assumption. While acknowledging the possibility of more effective ERAS protocols, it is crucial to note that such comparisons were beyond the scope of this study.

Notably, these protocols encompassed well-defined patient selection criteria and postoperative guidelines, ensuring compliance with the established ERAS guidelines. Our findings align with previous research that has indicated the safety of performing LSG as an outpatient procedure [3, 12–23]. Surve et al., in a multi-institutional study on outpatient SG, reported 30-day readmission, reoperation, and ER visit rates of 0.6%, 0.6%, and 0.1%, respectively [3]. The study presented the results of 3162 outpatient SGs. Billing and colleagues conducted a similar study to evaluate the safety and effectiveness of this procedure in an ambulatory surgical setting [17]. The study included 2534 patients. They found that the procedure can be performed safely in an ambulatory surgical center with low rates of complications and no mortalities within 30 days of surgery. Their short-term complication rate was 0.1%. However, their 30-day readmission (2.5%) and reoperation (1.2%) rates were slightly higher compared to those reported by Surve et al. in their article [3].

Studies have also compared outpatient and inpatient SG procedures, revealing comparable outcomes between the two approaches [26, 27]. Dreifuss et al. compared the readmission rates between same-day surgery cases and inpatient SG cases [14]. They observed that the readmission rates with these two approaches were statistically similar. Another study compared the outcomes of same-day SG surgery and inpatient SG surgery using the MBSAQIP data [30]. The same-day discharge group had 7825 patients, and the inpatient SG had 263,833 patients. They observed a 0% mortality rate in the same-day discharge group. The leak, bleeding, 30-day reoperation, and morbidity rates were similar between the two approaches. However, they observed that the 30-day readmission rates were higher in the same-day discharge group (3.3% vs. 2.7%). Despite this, there were no reported mortalities associated with the outpatient procedure. In the present study, the readmission rate was 0.2%.

The available literature shows the presence of several associated risk factors contributing to the augmented incidence of readmission and reoperation following outpatient SG. Dreifuss and colleagues recently conducted a study on same-day discharge SG identifying risk factors associated with patient readmission [14]. The study analyzed data from the Metabolic and Bariatric Surgery Accreditation and Quality Improvement Program (MSSAQIP) and included 14,624 patients in the final analysis. The 30-day reoperation rate was found to be 0.7%, whereas our study revealed a rate of 0.5%. The readmission rates observed by Dreifuss et al. were higher than those in the present study (2.9% vs. 0.2%). The study also highlighted several independent risk factors, including female sex, preoperative GERD, renal insufficiency, and intraoperative drain placement, which were associated with a higher rate of readmission following same-day discharge SG. In contrast, the present study observed that approximately 84% of the patients were females, and GERD was the second most common preoperative comorbidity (28%). However, only 8.2% of the patients received an intraoperative drain. Despite the independent risk factors identified by Dreifuss et al., our study reported lower readmission (0.2%) with only one case of readmission. Drawing definitive conclusions regarding the specific factors underlying the favorable outcomes observed in our study presents challenges due to unanalyzed variables and dissimilarities with the Dreifuss study. Regrettably, crucial variables such as the preoperative neutrophil-to-lymphocyte ratio, postoperative opioid utilization, and reinforcement techniques involving running sutures were not assessed, impeding our ability to precisely identify the contributory elements to the favorable outcomes [31, 32]. Nevertheless, it is noteworthy to acknowledge notable distinctions between the studies. The study by Dreifuss et al. featured a higher proportion of patients undergoing concurrent procedures (59% vs. 12%) and a greater prevalence of patients categorized with an ASA score of IV (2.4% vs. 0%). Furthermore, the inclusion of general surgeons as operating surgeons (0.6% vs. 0%), resident doctors (4.4% vs. 0%), and fellows (2.1% vs. 0%) in the Dreifuss study potentially contributed to the observed discrepancies in outcomes.

The impact of length of stay on outcomes of outpatient SG is a contentious issue. Prolonged LOS is linked to increased healthcare costs, decreased patient satisfaction, and potential complications such as hospital-acquired infections. Conversely, early discharge may be associated with increased postoperative complications, readmission, or reoperation risks. However, recent studies have suggested that same-day discharge is feasible and safe for selected patients undergoing SG [3, 12–23]. In our study, we observed a remarkably short length of stay of 7 h and 30 min in our cases. This study stands as the inaugural report in the literature on the duration of time spent in the surgical center. This holds significance as it provides a foundational reference for forthcoming studies in this domain.

Despite the short length of stay, we did not observe any unfavorable outcomes or increased rates of readmission or reoperation. Our results indicate that same-day discharge may be a practical choice for appropriately selected patients and is non-inferior to hospital-based surgeries in selected patients [14, 27, 33]. Nonetheless, careful patient selection, surgical technique, and postoperative care are critical for optimizing outcomes and mitigating complications.

Several studies have investigated the incidence and causes of reoperation after outpatient SG. The most common causes of reoperation reported in the literature include bleeding, leakage from the staple line, sleeve stricture, and bowel obstruction [32]. In the present study, a mere two patients (0.7%) needed reoperation. Of the aforementioned cases, one patient developed an intra-abdominal hemorrhage on the day of the initial operation. The other patient underwent a concomitant procedure during the primary LSG. Following the surgery, the latter patient presented to the ER with complaints of chest pain. A computed tomography (CT) angiography of the chest with IV contrast was conducted, revealing the recurrence of a small hiatal hernia, which required reoperation. Importantly, this reoperation did not stem from a complication associated with the primary LSG. Instead, it was needed by the recurrence of a hiatal hernia previously repaired during the initial LSG.

The comprehensive investigation of LSG performed in outpatient settings provides significant clinical insights into bariatric surgery. The study highlights the current evolution in this field, owing to the increasing adoption of minimally invasive techniques and outpatient procedures. The shift towards less invasive approaches and integrating ERAS protocols yields several benefits, such as decreased postoperative pain, shorter recovery periods, and reduced hospitalization durations, thus enhancing bariatric surgery’s overall patient experience and cost-effectiveness. Furthermore, a systematic analysis of the study’s postoperative 30-day mortality, morbidity, readmission, and reoperation rates demonstrates promising outcomes, which position outpatient LSG as a safe and viable option. The low incidence of complications, zero 30-day mortality, and minimal occurrences of reoperation or readmission rates suggest that adopting outpatient LSG can substantially reduce healthcare expenses, improve access to bariatric surgery, and maintain high standards of patient care. These findings can potentially transform the landscape of bariatric surgery by providing healthcare practitioners and patients with a compelling rationale for embracing minimally invasive techniques in outpatient settings, ultimately benefiting the healthcare system and individuals seeking obesity surgery.

This study, being the highest level of evidence securing funding for a prospective trial, is not aimed at establishing a distinction in postoperative complications; quite the contrary, it asserts the non-inferiority of outpatient LSG compared to the hospital-based counterpart. If this were designed as a prospective randomized trial with two groups to detect a 1% difference in bleeding between inpatient and outpatient settings at 30 days, the requisite sample size would exceed 7500 patients. The study is unequivocally constrained by financial limitations, resulting in a reduced patient accrual and insufficient power to detect differences in rare complications, with the caveat that additional similar studies are imperative to validate and confirm our findings.

While the present study has generated comprehensive findings, it is imperative to acknowledge and account for certain limitations when interpreting the results. The study’s main limitation pertained to its small sample size, which may have implications for accurately representing rare complications. However, the sample size of 365 cases surpasses the average number of annual procedures performed by a typical surgeon. Therefore, in the scenario where an adept surgeon executes low-risk SGs, a correspondingly low complication rate would be expected.

Despite our efforts to be meticulous in checking for errors, there remained a chance of human error in the manual data entry process, which could have affected our analysis’s accuracy and reliability. Therefore, it is essential to acknowledge this limitation and exercise caution when interpreting the study results. Despite our efforts to be meticulous in checking for errors, there still remained a chance of human error in the manual data entry process, which could have potentially affected the accuracy and reliability of our analysis. Therefore, it is important to acknowledge this limitation and exercise caution when interpreting the results of the study.

Another limitation of this study is the heterogeneity of ERAS protocols employed across different centers for their respective patients. Nonetheless, our findings indicate that this variability did not significantly influence the overall study outcomes or the duration of patients’ stay in the surgical center. Future investigations that adopt a more standardized approach to ERAS protocols are warranted to comprehensively elucidate the specific elements within these protocols that yield the most significant benefits for patient outcomes.

Additionally, including patients who underwent robotic-assisted SG represents a notable aspect of this study’s limitations. However, it is essential to highlight that this particular subgroup constituted a minor proportion of the study population, and the discharge criteria remained unaffected by the utilization of robotic surgery. Furthermore, it is essential to consider that all participating surgeons in this trial exhibited a high level of experience, having performed numerous SG procedures before their involvement. As a result, the similarity of outcomes across specialized centers in the United States, where highly experienced surgeons are prevalent, should be interpreted with caution when extrapolating to less experienced centers and surgeons. One limitation of our study is the need for more specific data on the number of patients who were discharged from the surgical facility to a monitored setting with clinical oversight, nurse availability, and access to intravenous fluids. This absence of information represents a constraint in our ability to provide detailed insights into the prevalence and impact of such postoperative care practices, thus highlighting a limitation of the study.

Despite the potential limitations of this multi-institutional study, its strength lies in its prospective design, which allowed for the collection of high-quality data on outpatient LSG. In the existing literature on outpatient SG, it is noteworthy that this study is the sole Level 2a evidence, contributing valuable insights to the field. Moreover, the involvement of 19 surgeons from six different centers enhanced the generalizability of the findings and minimized the impact of institutional biases. By collecting data from multiple centers, the study captured a wide range of patient characteristics and surgical techniques, thus increasing the external validity of the findings. Our study demonstrates another notable strength: Despite the absence of standardized surgical techniques, our findings align with and are comparable to the outcomes reported in the existing literature. At the same time, it acknowledges the significance of specific surgical techniques, including factors such as bougie size, distance from the pylorus, and staple line reinforcement. These factors may play a role in complications, and future studies should explore their influence to optimize surgical practices and enhance patient outcomes. Consequently, we emphasize the importance of future investigations to delve into these aspects further, aiming to optimize surgical practices and enhance overall patient outcomes.

Overall, this study’s prospective design and multi-institutional collaboration are significant strengths that contribute to the quality and generalizability of the results.

## Conclusions

In conclusion, this multi-institutional study demonstrated that experienced surgeons could safely perform outpatient LSG in a carefully selected patient population with low complication rates. This approach offers a cost-effective and expedient avenue for patient care while safeguarding the achievement of desired surgical outcomes. Our findings add to the growing body of literature supporting the idea that outpatient bariatric surgery is not inferior to hospital-based surgery and emphasize the importance of further research and exploration of this surgical setting.
